# Enhanced Delivery of Transforaminal Epidural Steroid Injection (TFESI) for Radiculopathy Associated with Acute Lumbar Disc Prolapse Through Physiotherapist-led Assessment, Consent, and Direct Listing

**DOI:** 10.5812/aapm-171054

**Published:** 2026-04-30

**Authors:** Greta Mattocks, Layla Meshykhi, Benjamin Thornton, Andreas Goebel, Martin Wilby, Canisius Dzapasi, David Woodward, Maneesh Bhojak, Manish Gupta

**Affiliations:** 1 Walton Centre Foundation Trust, Liverpool, United Kingdom; 2 Pain Research Institute, University of Liverpool, Liverpool, United Kingdom

**Keywords:** Epidural Injection, Physiotherapy, Radiculopathy, Lumbar Disc Herniation

## Abstract

**Context:**

Invasive treatments for lumbar radiculopathy with persistent symptoms and concordant disc prolapse include a transforaminal epidural steroid injection (TFESI) and microdiscectomy. Evidence supports TFESI as a potential first-line alternative to surgery. Our expedited nerve root block service (ERBS) reduced progression to surgery; however, waiting times remained suboptimal.

**Objectives:**

We evaluated a streamlined pathway incorporating physiotherapy-led consent and direct TFESI listing.

**Methods:**

This retrospective observational study evaluated a pathway modification in which the pre-procedure specialist consultation was removed. Two Musculoskeletal Clinical Assessment Service (MCAS) physiotherapists were trained to assess suitability, obtain consent, and list patients directly for TFESI, with consultant validation conducted virtually. The primary outcomes were the median waiting time from MCAS consent to TFESI and the change in waiting time compared with the previous pathway. Secondary outcomes were the proportion of listings validated, patient-reported response to TFESI, progression to surgery, and the association between TFESI response and disc pathology.

**Results:**

A total of 129 patients provided consent (mean age, 47.3 ± 12.7 years; 95% confidence interval (CI), 45.1 - 49.5; 63.6% female), with 100% validation. The median waiting time from MCAS consent to TFESI was 50 days (range, 2 - 262 days), representing a 37-day reduction (43%) compared with the previous pathway. A positive TFESI response occurred in 83% of patients, and 14% required microdiscectomy. Response rates were similar for disc protrusion (84%) and extrusion (81%).

**Conclusions:**

A physiotherapy-led ERBS pathway was associated with reduced waiting times and no identified safety concerns, while maintaining a low conversion rate to surgery.

## 1. Background

Lumbar radiculopathy associated with acute lumbar disc prolapse is common in the United Kingdom; an estimated 13% to 40% of individuals will experience at least one episode during their lifetime (1). Most patients show spontaneous improvement within 6 - 8 weeks of symptom onset with conservative management (2).

Management options for patients with persistent symptoms and symptom-concordant disc prolapse include pulse intravenous methylprednisolone infusion, Racz catheter techniques, epidural steroid injection, such as parasagittal interlaminar or transforaminal epidural steroid injection (TFESI), and surgical intervention in the form of microdiscectomy (3-5). The large randomized controlled NERVES trial demonstrated equivocal efficacy outcomes for TFESI and microdiscectomy, with a higher number of adverse events recorded after surgery; the authors therefore concluded that TFESI should be considered the first-line invasive treatment option (2). We consequently established an expedited nerve root block service (ERBS) at our tertiary-level center, which serves as the sole such service regionally (catchment area: North West England; population approximately 3 million) (6).

The service comprised 2 specialist spinal physiotherapists and a single pain consultant, who performed all procedures with a dedicated theater team. Triage criteria followed NERVES principles, prioritizing acute radiculopathic pain consistent with disc prolapse and concordant imaging (2). The ERBS pathway operated within the wider neurosurgical and spinal service and was aligned with neurosurgical pathways rather than functioning as a standalone pain service (6).

Audited outcomes highlighted the effectiveness of this service in clinical practice, with a substantial reduction in the number of surgical procedures (6).

We observed that the time between referral for and performance of TFESI was suboptimal (previously unreported median, 87 days, excluding pandemic-related delays). As there were no significant theater-scheduling constraints, our redesign targeted upstream delays rather than capacity.

## 2. Objectives

We conducted a retrospective observational evaluation of a quality-improvement initiative to assess whether a simplified ERBS pathway could reduce waiting times while maintaining the previously reported low rate of surgical intervention for lumbar radicular pain associated with symptom-concordant disc pathology (6).

## 3. Methods

Previously, Musculoskeletal Clinical Assessment Service (MCAS) clinicians (physiotherapists with subspecialization in spinal pathology) or consultant surgeons referred patients to a pain specialist for both consultation and consent for TFESI (6).

**Figure 1.**
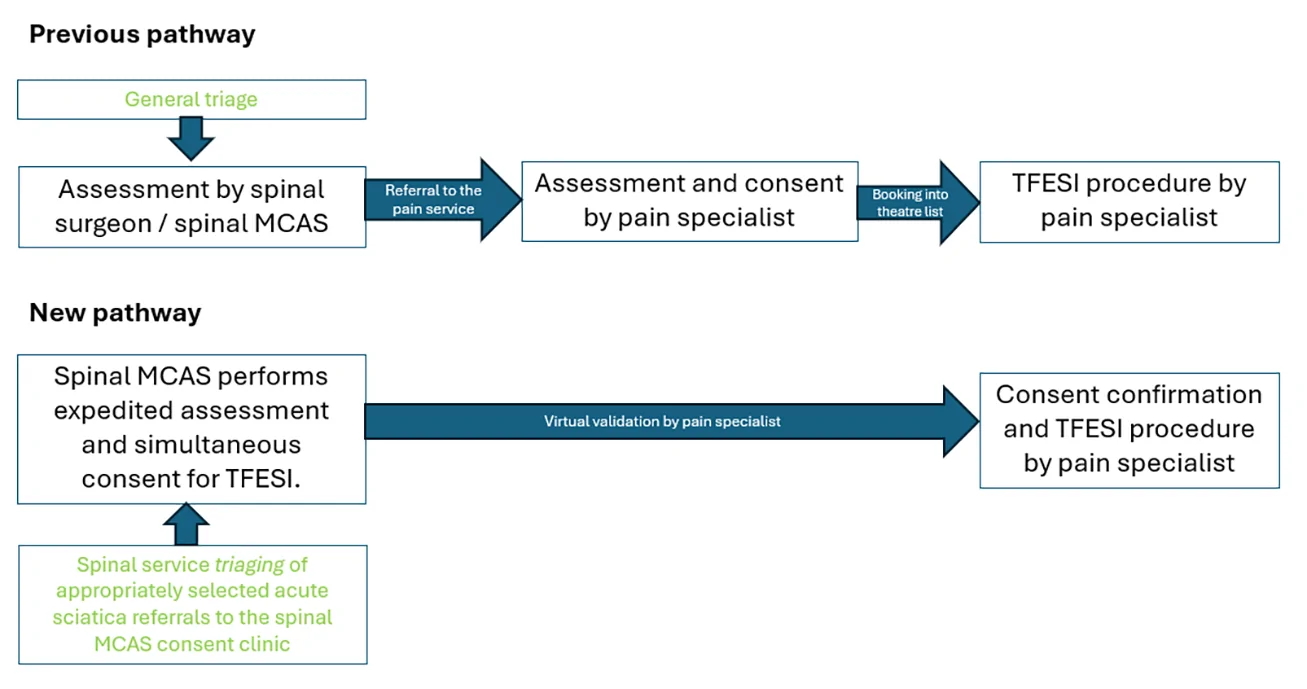
New pathway for Expedited Nerve Root Block Service (ERBS)

A key source of delay was a duplicate queue created by the consultant-led pre-procedure consent clinic, which required a separate specialist appointment (6). The pathway redesign targeted this duplication (Figure 1).

Two MCAS clinicians received additional training in assessing patients for TFESI suitability and obtaining procedural consent. Safety was maintained through defined inclusion and exclusion criteria, escalation pathways, and consultant oversight of final listing and procedure delivery (Appendix in the Supplementary File).

Following patient assessment and consent, MCAS clinicians directly listed suitable patients for the TFESI procedure. The operating pain clinician performed virtual validation between MCAS consent and the TFESI procedure date, with further confirmation of consent on the day of the procedure. Specialist review was conducted noncontemporaneously to minimize its effect on pathway duration. This process eliminated non-value-adding steps, including the need for a separate clinic and the associated wait, thereby shortening total waiting time while preserving clinical governance and safety.

We analyzed data from our hospital-internal referral database for all referrals made to the ERBS over an initial 27-month period from May 2021, when the new pathway began, through August 2023.

The comparator (previous pathway) was derived from our previously published evaluation of the ERBS service (6). For the current pathway, waiting time was defined as the interval from MCAS consent to the procedure. The previous 87-day waiting-time value from the prior evaluation was considered directly comparable, and patient cohorts were drawn from the same service population (6).

Inclusion and exclusion criteria for patients listed for TFESI largely resembled those of the NERVES trial and our previous work (2, 6), ensuring comparability of patient groups. There were no substantive changes in triage rules, staffing, or procedural practice since our previous work, conducted in 2019 - 2021, and no concurrent service modifications were identified that could affect waiting time. Patients were aged 16 - 65 years and had a diagnosis of sciatica (radiculopathy) associated with a pain-concordant centrolateral herniated lumbar disc, with pain that had not responded to conservative noninvasive management (medications and/or watchful waiting, with physiotherapy input in some patients). Whereas patients accepted for the cited trial had symptom durations strictly between 6 weeks and 12 months, we also accepted patients with symptom durations somewhat longer than 12 months (2). Patients were not listed if they had signs of significant neurological deficit (motor weakness or possible cauda equina syndrome) or were known to be pregnant. All block procedures were performed by the same consultant pain specialist.

All data were retrospectively extracted by a single clinician from complete clinical records, including follow-up correspondence and MRI reports, thereby avoiding duplicate extraction. Reporting reliability was supported by focused training from a consultant neuroradiologist and independent verification of a sample of MRI findings and outcome classifications by both a neuroradiologist and a senior pain consultant. Missing or ambiguous documentation was prespecified as missing and excluded from subgroup analyses rather than inferred.

We recorded patient age, sex, and symptom duration as reported at the initial patient encounter. We documented whether MCAS listings remained valid following consultant virtual review and recorded the time from MCAS consent to the TFESI procedure, which was the primary waiting-time outcome.

The patient-reported TFESI outcome was also recorded from follow-up clinic letter entries for clinics scheduled 6 - 8 weeks after the procedure, when patients were routinely seen by the same MCAS clinical assessor who had previously consented and listed them. TFESI outcomes were documented similarly to the previous audit study (6). Briefly, outcomes were dichotomized as positive or negative. A positive outcome was defined as clinical improvement at follow-up without the need for further escalation of care (eg, surgical referral or additional specialist intervention) and patient-reported meaningful pain relief, irrespective of duration.

Intervertebral disc pathology was recorded from MRI reports. We also determined whether the patient was referred for or underwent subsequent spinal surgery within the observation period, with follow-up through August 2025, 2 years after the last MCAS consent.

The primary outcomes were 1) median waiting time from MCAS consent to the block procedure and 2) change in waiting time compared with that recorded before pathway redesign. Secondary outcomes included the percentage of MCAS consents virtually validated by the pain consultant (Figure 1), the rate of progression to surgery (microdiscectomy), and, because pathologies other than disc protrusion had previously been associated with a lower block success rate, the relationship between block success and specific disc pathology (6).

Balancing measures and safety were assessed alongside the primary outcomes to identify unintended consequences of the pathway redesign, including incorrect patient listing, failures to detect contraindications, rebookings, adverse events, complaints, or procedure-day cancellations due to consent or eligibility issues (Appendix in the Supplementary File).

## 4. Results

A total of 129 patients were consented by MCAS clinicians over the study period (mean age, 47.3 ± 12.7 years; 95% CI, 45.1 - 49.5; 63.6% female), and 100% were virtually validated by the pain consultant. A total of 112 TFESI procedures were performed. Seventeen patients (13.2%) canceled the procedure before the listing date, and 58.8% of these cancellations were documented as being due to spontaneous symptom improvement.

The median waiting time from MCAS consent to the procedure was 50 days (range, 2 - 262 days), representing a reduction of 37 days (43%) compared with our previous results (6). Comparison of waiting times between the first and second halves of the evaluation period showed that the reduction was sustained, with possible further improvement. A total of 13% of injected patients waited longer than 100 days, most commonly because of delays in obtaining patient documentation (Appendix in the Supplementary File). Only 14% of consented patients subsequently underwent microdiscectomy by the August 2025 cutoff.

*Positive outcomes* from TFESI were documented in 83% of patients. Among patients with available imaging (n = 107/112), 65.4% (70/107) had intervertebral disc protrusion, 33.6% (36/107) had disc extrusion, and 0.9% (1/107) had sequestration.

*Positive outcomes* were reported in 84% of patients with disc protrusion and 81% of those with disc extrusion.

For this clinical service, some patients were included with a symptom duration longer than 12 months. We assessed whether outcomes differed between patients within and outside the original 12-month inclusion criterion for the NERVES trial (2). Symptom duration was available for 84.5% (109/129) of consented patients; of these, 17.4% (19/109) reported symptoms lasting 12 months or longer (n = 13 for < 18 months; n = 6 for > 24 months, predominantly due to pandemic-related pathway delays). Of the 19 consented patients with pain lasting longer than 12 months, 18 (94.7%) received TFESI, and 1 patient canceled the procedure.

Among patients who received TFESI and had follow-up data, 81.3% (13/16) reported a positive outcome; 2 patients could not be contacted. Of patients with symptom duration ≥ 12 months, 10.5% (2/19) subsequently underwent microdiscectomy.

## 5. Discussion

The establishment of a streamlined, MCAS-based referral and consent pathway for ERBS was associated with a safe and effective service. Our data suggest that training MCAS clinicians (Appendix in the Supplementary File) resulted in high-quality TFESI listings; the consultant pain specialist proceeded with TFESI for all listed patients who attended. This high quality was subsequently sustained: among MCAS-consented patients listed between August 2023 and August 2025, after the inclusion cutoff for this study, only 1 procedure listing was not validated by the consultant because information on diabetic control was missing. MCAS retraining in this aspect was subsequently performed (data not shown).

The new pathway achieved a substantial reduction in waiting time for treatment compared with the previous pathway (median, 87 vs 50 days; relative reduction, 43%) (6). Although standards for acceptable waiting times for TFESI for this indication have not been established, and data on rates of natural improvement over time in this patient group are limited, a median wait of 50 days (approximately 7 weeks) appears acceptable. This timeframe limits the duration of suffering from this painful condition while also recognizing its frequently benign natural course (1). Further efforts are required to reduce the proportion of outliers with waiting times longer than 100 days (currently 13%).

Similar to our previous work, the new pathway again achieved a very low conversion rate to subsequent surgery, consistent with the NERVES trial results (2, 6).

Unlike our previous study (6), we observed no differences in TFESI outcomes by disc pathology. We also observed acceptable outcomes in patients with longer symptom duration (> 12 months).

### 5.1. Study Limitations

A limitation of our study is that we did not capture patient waiting times to MCAS assessment. Greater awareness among referrers and patients of the value of TFESI may help minimize this interval.

Because this evaluation was retrospective, detailed measures such as pain scores, function, analgesic use, and return to work were not consistently available at 6 - 8-week follow-up. Outcomes were therefore derived from clinic correspondence. Although this approach may introduce misclassification bias, it was applied consistently across both the present work and our previous study, supporting internal validity (6).

Furthermore, this was a single-center study in which MCAS training was limited to 2 clinicians, and all procedures were performed by a single consultant. Although this enhanced procedural consistency, it may have reduced external validity and reflects real-world service-delivery constraints; therefore, data from other settings would be valuable to confirm these findings.

### 5.2. Conclusions

This evaluation demonstrates that a streamlined ERBS pathway incorporating MCAS-led listing was associated with reduced waiting times without identified safety concerns. Although causality cannot be inferred because of potential confounding factors, including temporal changes in demand, scheduling capacity, and seasonal variation, these findings support the feasibility of combining MCAS-led consent with consultant validation. This approach may be transferable to centers with similar service configurations and warrants prospective evaluation.

aapm-16-2-171054-s001.pdf

## Data Availability

The dataset presented in the study is available on request from the corresponding author during submission or after publication. The data are not publicly available due to requirements for maintaining patient confidentiality and compliance with NHS information governance standards.
