# Supplementary material for: Enhanced Delivery of Transforaminal Epidural Steroid Injection (TFESI) for Radiculopathy Associated with Acute Lumbar Disc Prolapse Through Physiotherapist-led Assessment, Consent, and Direct Listing
Source: Anesth Pain Med. 2026 Apr 30;16(2):e171054. doi: 10.5812/aapm-171054 (PMC13589457; doi:10.5812/aapm-171054)
Supplement: Supplementary file 1 [file aapm-16-2-171054-s001.pdf]

Appendix:

Consent training and pathway implementation:

Spinal MCAS practitioners, already skilled in MRI interpretation and decision-making for surgical or TFESI, underwent real-time pain clinic training (three sessions) with emphasis on consent policies and clinical governance.

Following this, a Standard Operating Procedure (SOP) was established and approved by the Director of Pain Services, the Spinal Surgery Lead, and the hospital Clinical Governance Lead.

Eligibility:

Medically non-complex patients presenting with classic sciatica symptoms and corroborating MRI findings.

Minimum documentation requirements in the clinic letter:

- Current medications, including allergies
- Past medical history
- MRI findings (including any transitional levels if reported) and symptoms
- Full consent discussion of risks and benefits, including: Alternative treatment options and non-intervention (wait-and-watch approach)

Process:

- MCAS practitioners triage appropriate patients from the spinal pool and obtain consent during clinic visits
- Defined exclusion criteria applied at point of selection (
- All consented cases undergo electronic validation by the pain consultant prior to the procedure date, including review of imaging and accessible primary and secondary care records

Implementation:

- Regular email discussions and meetings between MCAS practitioners and the pain consultant refined the pathway
- Administrative teams in spinal and pain services were trained to support communication and listing

- Ongoing feedback supported implementation

Safety:

Predefined adverse outcome measures:

- Incorrect or inappropriate listing
- Failure to identify contraindications
- Same-day cancellations due to eligibility or consent issues;
- Re-bookings
- Documented adverse events
- Patient complaints.

Data sources:

- Electronic patient record
- PAS (appointment outcomes, cancellations, re-bookings)
- procedure logs (completed vs abandoned procedures)
- Clinic correspondence (eligibility and consent issues)
- Institutional incident reporting system (DATIX) for safety events and near-misses.

Safety processes:

- Mandatory consultant validation of all patients consented and listed by MCAS clinicians
- Routine pre-procedure review of clinical records and imaging.
- This provided multiple checkpoints to detect contraindications, incorrect selection, or inappropriate listing.

- **Verification** with routine clinical governance processes and procedural outcome review

Outcome findings:

No safety events or balancing measure failures were identified during the study period. Minor procedural events, such as infrequent vasovagal episodes, were recognised as expected transient reactions consistent with standard clinical practice and did not result in harm or require escalation of care. No major adverse events, including neurological or infective complications, were observed, and there were no cases of procedure-related morbidity requiring further intervention.

Consent

This work was undertaken as a service evaluation/clinical audit and received formal approval from The Walton Centre NHS Foundation Trust Clinical Audit Department (reference code provided in the manuscript).

As it involved retrospective analysis of routinely collected, anonymised clinical data, individual patient consent for participation or data use was not required in accordance with NHS Health Research Authority guidance.

All patients undergoing injections provided standard clinical consent for the procedure in line with Trust policy. This was separate from, and not related to, the use of data for audit purposes.

Waiting time analysis:

Median waiting time reduced from 54.5 days in the first half of the observation period to 39 days in the second half, indicating sustained improvement over time.

A more clinically relevant metric was adopted, defining extreme outliers as waits exceeding 100 days, as outlined below.

Waiting time from consent to procedure (days)	1st Half (observation period)	2nd Half (observation period)
Median	54.5	39
Range	9-197	2-262
Upper Bound (IQR)	171.13	121.38
Outliers	1	3

Delays in consent to procedure (>100 days):

Reason and number of patients who had a delay of more than 100 days from consent to procedure.

Category	n
Intercurrent/other medical interventions	4
Patient cancelled first offer	2
Awaiting relevant medical documentation	4
Difficulty contacting patient	1
Unclear	5
Total	16
